# National Veterinary College

**Published:** 1895-05

**Authors:** 


### NATIONAL VETERINARY COLLEGE.

The commencement exercises of the National Veterinary College were held on Saturday evening, April 6th. The address to the students was delivered by Edwin P. Willits. Dr. D. E. Salmon, President of the college, delivered the diplomas to the graduates, and conferred upon Dr. Charles F. Dawson and Dr. George Jobson, two of the faculty, the degree of D.V.S. Gold medal prizes for the best examination were awarded George E. Fetter, of New Jersey, while honorable mention was made of M. Wooden, of Maryland; William Fink, of Maryland, and Richard T. Mumma, of Pennsylvania. The junior anatomy prize was awarded J. W. Petty, of North Carolina. The degree of D.V.S. was conferred upon the following graduates: C. H. Blemer, of Kentucky; F. S. Charter, of Connecticut; J. M. Courbright, of Pennsylvania; A. L. Cundall, of Connecticut; T. H. F. Diederich, of Indiana; N. K. Fegley, oj Pennsylvania; G. E. Fetter, of New Jersey; William P. Fink, of Maryland; C. F. Hadfield, of Rhode Island; R. T. Mumma, of Pennsylvania; C. J. Siegmund, of Maryland; S. J. Swift, of Pennsylvania; F. W. E. White, of Virginia; M. D. Williams, of New York, and M. Wooden, of Maryland.

